# Speaker Accent Modulates the Effects of Orthographic and Phonological Similarity on Auditory Processing by Learners of English

**DOI:** 10.3389/fpsyg.2022.892822

**Published:** 2022-05-19

**Authors:** Candice Frances, Eugenia Navarra-Barindelli, Clara D. Martin

**Affiliations:** ^1^Basque Center on Cognition, Brain and Language (BCBL), Donostia, Spain; ^2^Department of Social Sciences and Law, The University of the Basque Country (UPV/EHU), Donostia, Spain; ^3^Max Planck Institute for Psycholinguistics, Nijmegen, Netherlands; ^4^Ikerbasque, Basque Foundation for Science, Bilbao, Spain

**Keywords:** bilingualism, auditory processing, cognates, phonology, orthography, lexical decision, typing

## Abstract

The cognate effect refers to translation equivalents with similar form between languages—i.e., cognates, such as “band” (English) and “banda” (Spanish)—being processed faster than words with dissimilar forms—such as, “cloud” and “nube.” Substantive literature supports this claim, but is mostly based on orthographic similarity and tested in the visual modality. In a previous study, we found an inhibitory orthographic similarity effect in the auditory modality—i.e., greater orthographic similarity led to slower response times and reduced accuracy. The aim of the present study is to explain this effect. In doing so, we explore the role of the speaker's accent in auditory word recognition and whether native accents lead to a mismatch between the participants' phonological representation and the stimulus. Participants carried out a lexical decision task and a typing task in which they spelled out the word they heard. Words were produced by two speakers: one with a native English accent (Standard American) and the other with a non-native accent matching that of the participants (native Spanish speaker from Spain). We manipulated orthographic and phonological similarity orthogonally and found that accent did have some effect on both response time and accuracy as well as modulating the effects of similarity. Overall, the non-native accent improved performance, but it did not fully explain why high orthographic similarity items show an inhibitory effect in the auditory modality. Theoretical implications and future directions are discussed.

## Introduction

As has been stated repeatedly in the literature, bilinguals are not “two monolinguals in one” (Grosjean, 1989, 1997, 1998; Grosjean and Nicol, 2007). There is ample evidence that a bilinguals' two languages interact in many ways (Caramazza and Brones, 1979; Grosjean, 2001; Lagrou et al., 2011; Blumenfeld and Marian, 2013). One common evidence of this interaction is the cognate effect (Caramazza and Brones, 1979; Cristoffanini et al., 1986; de Groot and Nas, 1991; Sanchez-Casas et al., 1992; Dijkstra et al., 1998, 1999; Schwartz et al., 2007; Voga and Grainger, 2007). The cognate effect refers to words that are similar in form and meaning between a bilingual's languages, activating the non-target language and thus having a processing advantage over words that only share meaning but not form—i.e., non-cognates or low similarity items. For example, “band” and “banda” are considered cognates between English and Spanish and would thus have an advantage over “cloud” and “nube”—non-cognates or low similarity items. One important distinction to make is that between orthographic/phonological cognates and “false cognates,” meaning interlanguage homographs and homophones that do not align in meaning. For example, <once> means “eleven” in Spanish, but in English it means “one time.” False cognates, given their semantic misalignment, do not share the same facilitatory effects of cognates in recognition (Dijkstra and van Heuven, 2002) or translation (Janke and Kolokonte, 2015). The cognate effect is quite well described with respect to orthographic cognates—namely, words that are orthographically similar between languages—leading to several processing benefits (van Orden, 1987; Duyck et al., 2007; Van Assche et al., 2011, 2012; Poort and Rodd, 2017). Given the strong cognate effect observed in bilingual speech and the common assumption that the two languages of a bilingual are co-activated at the phonological level (Costa et al., 2000; Colom, 2001; Colomé and Miozzo, 2010; Sadat et al., 2016), words that are phonologically similar between languages may also influence word processing.

In a prior study on the effect of phonological similarity on lexical processing, we found that there was an inhibitory orthographic similarity effect in the auditory modality (Frances et al., 2021). This study showed that, with greater orthographic similarity between the spoken word in the native (NL) and foreign (FL) languages, response times were slowed and accuracy was reduced. For example, when Spanish-English bilingual participants heard the English word “band,” which is both a phonological and an orthographic cognate (“banda” in Spanish), their responses were slower than when they heard “jacket”(/dʒækət/), which is a phonological cognate but an orthographic non-cognate (“chaqueta” pronounced /t∫aketa/ in Spanish). In addition, other studies have found similar cross-modality inhibition [e.g., phonological inhibition in the visual modality (Dijkstra et al., 1999; Lemhöfer and Dijkstra, 2004)]. These results point to an independence but co-activation of representations in both modalities. Not only that, but the cross-modal inhibition suggests that the particular relationship between orthography and phonology (i.e., whether they generally have a one-to-one correspondence or not) in each of the languages of a bilingual can influence the cognate effect.

One possible way of explaining this inhibitory orthographic effect in the auditory modality is through a discrepancy between the listener's (FL) phonological representation of the FL item and the native speaker's production of it. In other words, the listener's NL is likely to affect not only their production of FL words, but also their internal phonological representation of them. In addition, individuals with a transparent—i.e., a language with a one-to-one correspondence between the graphemes and the phonemes—NL are likely to have a stronger reliance on orthography than those with an opaque NL, as orthographic consistency aids the auditory processing of words (Seidenberg and Tanenhaus, 1979; Ziegler and Ferrand, 1998). When the participant's NL is transparent, this distortion is likely to be greater, with more interference in items that are orthographically similar between languages. If that is the case, hearing an orthographic cognate said by a native speaker is likely to mismatch with the FL listener's representation of the item. This would, in turn, slow down the process of verifying that the item is in fact a real word. In language production, there are studies that found increased accentedness in the production of orthographic cognates in FL speakers (Costa et al., 2000; Amengual, 2012; Goldrick et al., 2014). This provides support for the idea that cognates may suffer from a greater phonological influence of the NL. Therefore, for instance, Spanish-English bilinguals would have a stronger Spanish accent when producing “band” as compared to “jacket,” with “band” being an orthographic cognate, whereas “jacket” is not. Given that the foreign accent is stronger, it might be the case that the internal representation of “band” is more strongly influenced by grapheme-phoneme correspondence rules in Spanish than that of “jacket.” In other words, the native Spanish listener's internal representation of the English word <violin> is /biolin/ because of the orthographic similarity with the Spanish translation <violín>. When they hear /vaɪəˈlɪn/, there is a strong mismatch between the perceived word and its internal representation, making word recognition slower.

Another possible way to explain the inhibitory orthographic effect in the auditory modality is that the auditory stimuli activate incorrect orthographic representations. Speakers often rely on orthography to process phonological items (Seidenberg and Tanenhaus, 1979; Ziegler and Ferrand, 1998), possibly “transcribing” the phonological string into its orthography when doing an auditory lexical decision task (LDT). This is particularly problematic in the case of bilinguals with a transparent NL and an opaque FL, as their NL rules are likely to influence and distort this process. For example, when the participant hears the English word <violin> (/vaɪəˈlɪn/), they transcribe it as <baiolin> using their NL phoneme to grapheme correspondence rules, which is quite different from the correct FL spelling of the word (<violin>). When they hear the word <jacket> (/dʒækət/), they transcribe it into something like <jaket>, which is much closer to the correct orthography for the item in English (<jacket>).

The main aim of the present study is to explain the inhibitory orthographic similarity effect in the auditory modality by testing whether it is due to a mismatch (1) between the internal phonological representation and the aural stimulus or (2) between the constructed and real orthographic representations, or (3) possibly a combination of the two. In addition, we will explore the effects of accent—native vs. foreign—in both detecting and identifying words as well as whether and how this interacts with orthographic similarity between languages.

To test this question, the current study includes the following tasks: (1) an LDT and (2) a typing task, both in the auditory modality. In the auditory LDT, we approximate participants' internal phonological representation (in their FL) by presenting stimuli in the accent that is closest to theirs and the pronunciation they are most accustomed to, produced by a non-native speaker with the same origin (as well as a native speaker as a control condition). For the typing task, participants simply type what they hear (phonological strings including words and pseudowords) when they are presented with the auditory stimuli—produced by both the native and non-native speakers.

Based on the two possible explanations we have presented, there are different expected results. One option is that our first explanation is correct and the inhibitory effect of orthographic similarity is due to the discrepancy between the internal phonological representation and what the FL listeners are hearing. This would also mean that bilinguals have particularly accented representations of orthographic cognates. If that is the case, (1) participants would show the inhibitory orthographic effect only when they hear the words in the native accent, but not in the non-native accent. In other words, by hearing words in the non-native accent, the stimuli would match their internal representations of the phonological items more closely, thus negating the inhibitory effect of orthographic similarity. On the other hand, in the typing task, participants have extra time to process the stimuli. Therefore, they are unlikely to show the orthographic inhibitory effect in neither the native nor non-native accent. In fact, they are likely to show a facilitatory effect of orthography, as the NL orthography should aid spelling in cases of high similarity. The other option is that our second explanation is correct. In this case, the inhibitory orthographic effect would be due to a mismatch between the constructed and real orthographic representations during aural perception. If so, (2) we would expect to see the inhibitory effect of orthographic similarity in both accents in the LDT. The idea is that, if the phoneme to grapheme correspondence between the NL and FL is the cause of the effect, then the effect should remain unaffected by accent, as this correspondence does not depend on production. We should also see it in the typing task, with an increase of typing errors in orthographically similar words.

In other words, what makes the largest difference between the two hypotheses is that in the first case (1) the inhibitory orthographic similarity effect should disappear in the LDT with the non-native accent and we should see a facilitatory effect of orthographic similarity in spelling in the typing task, whereas in the second case (2) the inhibitory orthographic similarity effect should not disappear in the LDT regardless of accent and there should be an inhibitory orthographic effect in the typing task, as well. In addition, we expect higher performance overall with the non-native accent (see interlanguage speech intelligibility benefit) (Bent and Bradlow, 2003; Xie and Fowler, 2013; Wang and van Heuven, 2015).

## Methods

### Participants

Participants were 59 native Spanish speaking adults (*F* = 37, M_age_ = 27.86 [SD = 4.42]) from Madrid and Murcia (Spain) with at least an intermediate (B1) level in English. Participants had a minimum score of 40 on the English BEST (de Bruin et al., 2017)—a picture naming task with a maximum score of 65—and of 55% on the English LexTALE (a vocabulary test), which equates to approximately a B2 (upper intermediate) level (Lemhöfer and Broersma, 2012). Participants' average score on the BEST was 60.88 (SD = 4.95) with a range of 42–65. With respect to the LexTALE, their average score was 76.41% (SD = 10.35%) with a range of 55–99%. Their average self-reported age of acquisition of English was 6.61 (SD = 3.08) years old, with a range of 3–19 years of age. All participants provided informed consent before taking part in the experiment, which was conducted in accordance with the Declaration of Helsinki and approved by the Basque Center on Cognition, Brain and Language ethics committee (approval number 12762). Participants were paid for taking part in the experiment.

### Stimuli

**Figure 1:**
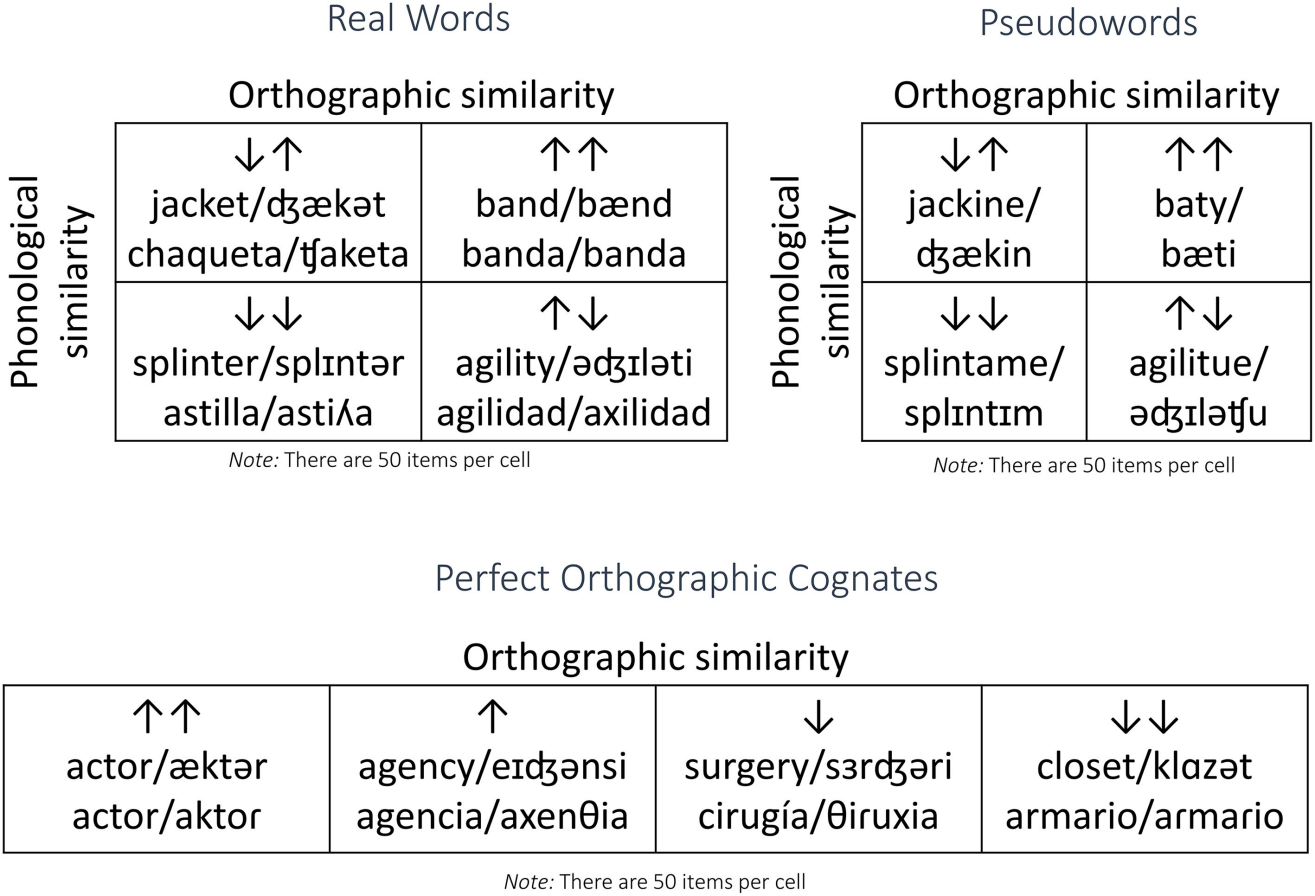
Example stimuli in each similarity condition.

Stimuli consisted of 300 English words and 300 pseudowords. The words were taken from Frances et al. (2021). These were divided into six categories (see Figure 1). Four of them consisted of a Latin square between orthographic and phonological similarity: high orthographic/high phonological similarity, high orthographic/low phonological similarity, low orthographic/high phonological similarity, and low orthographic/low phonological similarity. In our case, the terms “high similarity” and “low similarity” were favored over “cognate” and “non-cognate” because the distribution of similarity we based this distinction on is linear rather than dichotomous. The categories of high and low similarity were determined using a median split of ALINE distance (Kondrak, 1999, 2000).

Another group contained items in the extreme of the orthographic similarity distribution: orthographically identical words or perfect cognates. Finally, we included a group of extreme dissimilarity items in order to balance the number of high and low similarity items (see Figure 1 for an example of each).

As mentioned above, high and low phonological and orthographic similarity were defined by median split using inverse ALINE distance (Kondrak, 1999, 2000). ALINE distance is a normalized measure of string alignment that provides a value of dissimilarity (with inverse ALINE distance being a measure of similarity) between words. This can be used to compare translations between languages. For phonology, we placed the median split at 0.740. The high similarity range of inverse ALINE distance values was 0.741–0.951 and for the low similarity group, the range was 0.195–0.736. For orthography, we used the median split was at 0.770. The high similarity range of inverse ALINE distance values was 0.771–0.982 and for the low similarity group, the range was 0.360–0.769. ALINE distances were calculated using the alineR package for R (Downey et al., 2017).

For the main manipulation, we focused on the first four groups. The orthographically identical translation group (perfect orthographic cognates) were included to assess the “special status” of those words, also called “perfect cognates.” Note that no phonologically identical group was included, since the differences in phonology between English and Spanish made it impossible to find enough items.

All six groups of items were matched on the following variables: word frequency (raw and logarithmic), word frequency of the Spanish translation (raw and logarithmic), number of syllables, number of letters, and number of phonemes (see Table 1 for means, standard deviations, and statistics), all extracted from CLEARPOND (Marian et al., 2012). Pseudowords were created by exchanging the last two phonemes (2 or 3 letters) between words used in the task (e.g., lens/lεnz changed to lert/lεrt and airport/*εrpɔrt to airpons*/εrpɔnz). This way, the number of letters and phonemes remained constant and all items had to be listened up to the penultimate phoneme in order to differentiate the word from the pseudoword. In other words, we maintained the uniqueness point of target words constant between stimuli and as late as possible.

**Table 1:** Means, standard deviations, and statistics for variables stimuli were matched on.

**Orthographic similarity**	**Low**		**High**		**Identical**	
**Phonological similarity**	**Low**	**High**	**Low**	**High**	**High**	**Statistic**
English frequency	25.21 (21.85)	30.81 (43.55)	42.61 (80.29)	24.63 (21.65)	31.64 (49.27)	*F*(4,245) = 1.117, *p* = 0.349, *BF*_01_ = 16.175
English log frequency	1.22 (0.45)	1.12 (0.61)	1.27 (0.57)	1.23 (0.40)	1.17 (0.51)	*F*(4,245) = 0.667, *p* = 0.616, *BF*_01_ = 32.943
Spanish frequency	50.37 (56.59)	67.63 (80.91)	79.34 (106.46)	58.89 (58.58)	69.22 (101.77)	*F*(4,245) = 0.864, *p* = 0.486, *BF*_01_ = 24.147
Spanish log frequency	1.38 (0.63)	1.47 (0.65)	1.47 (0.69)	1.48 (0.64)	1.38 (0.72)	*F*(4,245) = 0.304, *p* = 0.875, *BF*_01_ = 58.305
Number of syllables	2.00 (0.88)	2.08 (0.92)	2.00 (0.90)	1.86 (0.76)	1.88 (0.39)	*F*(4,245) = 0.670, *p* = 0.613, *BF*_01_ = 32.769
Number of letters	6.36 (1.96)	6.08 (2.06)	6.52 (1.76)	6.38 (2.00)	5.84 (1.17)	*F*(4,245) = 1.126, *p* = 0.345, *BF*_01_ = 15.940
Number of phonemes	5.94 (2.08)	5.98 (2.20)	5.46 (1.76)	5.88 (1.83)	5.56 (1.07)	*F*(4,245) = 0.839, *p* = 0.502, *BF*_01_ = 25.110

*Values are means with standard deviations in parentheses. The N in all cases is 50*.

There was a total of 50 words per group, for a grand total of 300 words. There were also 50 pseudowords per condition—one matched to each word. All words and pseudowords were presented once in each accent condition (see below).

Native accent auditory stimuli were recorded in a quiet recording room by a native speaker of English with a general American accent (Labov et al., 2006) and following the pronunciation reported in the Carnegie Mellon CMU dictionary (Carnegie Mellon, 2020). Foreign accented (non-native speaker) auditory stimuli were similarly recorded by a native speaker of Spanish. Importantly, the non-native speaker did not add or remove phonemes to the words, they simply used the closest Spanish phoneme. For example, for <jacket> they produced /ʝaket/ instead of /ʤækət/ and for <violin> they produced /baiolin/ instead of /vaɪəˈlɪn/. All stimuli were normalized to 1dB and cut with 500 ms of silence before and after, using Audacity (Audacity Team, 2018). They were recorded at a frequency of 44.1 kHz and 32 bits.

### Procedure

Participants were pre-selected using a form in which they reported age, language background, and were tested on their level of English [BEST (de Bruin et al., 2017) and LexTALE (Lemhöfer and Broersma, 2012)]. This was completed using LimeSurvey (Schmitz LPT/C, 2019). The stimuli in the testing sessions were presented using Opensesame (Mathôt et al., 2012) through the JATOS platform (Lange et al., 2015).

The data collection consisted of two sessions: one with the native accented stimuli and the other with the non-native accented stimuli. The order of sessions was counterbalanced between participants, and there were at least 2 weeks between the two sessions. In each session, participants first carried out an LDT and then a typing task, with the same stimuli. For the LDT, participants were presented with all 600 items randomly mixed. For each trial, they would see a fixation cross for 500 ms, then hear the word and have 2,500 ms from stimulus onset to respond whether it was a real word or not using the F and J keys on the keyboard (counterbalanced between participants). Participants were provided a self-paced break every 150 words. After the LDT, they carried out the typing task. For the typing task, participants would have a fixation cross for 500 ms, then they heard the item twice. After the first utterance of the word, they were presented with a textbox in order to type in the item. They had unlimited time to type and they could erase and retype freely. The stimulus recordings were the same for the LDT and the typing task; in one session they were both native-accented and in the other they were both non-native-accented.

## Analysis

### Lexical Decision Task

The duration of each sound file was subtracted from the corresponding response times. Outliers were defined as values two standard deviations from the mean for each condition in each participant. In total, 4.55% of data was removed due to outliers. In all response time analyses, only correct responses were taken into account.

Data was analyzed categorically—low and high similarity as well as low similarity, high similarity, and perfect cognates—using ANOVAs. Accuracy was assessed using A', a measure of signal detection (Zhang and Mueller, 2005), calculated using the Psycho package (Makowski, 2018) for R. This measure was favored as it takes into account participant response tendencies. For A', all analyses were by participant, as we could not carry out by item analyses using A'—participants cannot be paired up as the stimuli were, to provide measures of correct rejections and false alarms.

Analyses were run using JASP (JASP Team, 2020). Additional analyses evaluating orthographic and phonological similarity linearly are provided in the Supplementary Materials.

### Typing Task

Due to technical errors, two participants had a reduced number of trials: one had 584 trials out of 600 for Day 1 and another had 514 out of 600 for Day 2. Finally, one participant had to be excluded because he was missing all of the typing task data for Day 1, leaving 58 participants—two of which had partial data. For this task, we carried out the same analyses as with the LDT: a three-way ANOVA (accent by orthographic similarity by phonological similarity) and a two-way ANOVA (accent by orthographic similarity including perfect cognates). Accuracy was defined as the number of correctly typed items (i.e., correctly identified and with no typos or spelling errors). All analyses were also run as linear models using ALINE distance (Lange et al., 2015) instead of the binary accuracy. Given that the pattern and significant effects and interaction were strictly identical, we omitted these from the main text. Additional analyses evaluating orthographic and phonological similarity linearly are provided in the Supplementary Materials.

## Results

### Lexical Decision Task

#### Phonological and Orthographic Similarity Effects on Response Time

**Figure 2:**
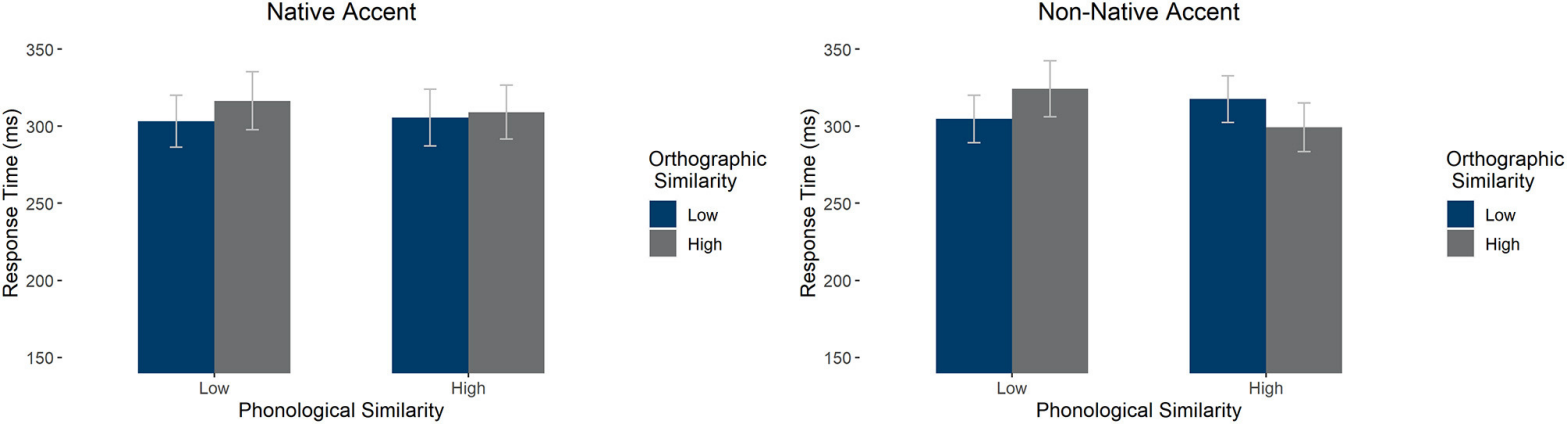
Average response times in the LDT by accent, orthographic similarity, and phonological similarity condition. Error bars mark 95% confidence intervals.

We carried out a two accent (native/non-native) by two orthographic similarity (high/low) by two phonological similarity (high/low) repeated measures ANOVA on response times. There was a significant interaction between orthography and phonology [*F*_1_(1,58) = 14.296, *p* < 0.001, $\eta_{\text{p}}^{2}$ = 0.198, absent in by item analysis *F*_2_(1, 196) = 1.597, *p* = 0.202, $\eta_{\text{p}}^{2}$ = 0.008] as well as a three-way interaction between accent, orthography, and phonology [*F*_1_(1,58) = 8.834, *p* = 0.004, $\eta_{\text{p}}^{2}$ = 0.132, absent in by item analysis *F*_2_(1, 196) = 0.348, *p* = 0.556, $\eta_{\text{p}}^{2}$ = 0.002]. See Figure 2 for average response times. We observed a main effect of accent (slower for non-native) only in the by item analysis [*F*_1_(1,58) = 0.302, *p* = 0.585, $\eta_{\text{p}}^{2}$ = 0.005; *F*_2_(1, 196) = 29.484, *p* < 0.001, $\eta_{\text{p}}^{2}$ = 0.131]. There were no other significant effects (all p's > 0.1)

Follow-up two-way ANOVAs exploring phonological and orthographic similarity effects independently for native and non-native accent showed different effects in the two accent conditions. In the native accent, there was a main effect of orthography [*F*_1_(1,58) = 5.522, *p* = 0.022, $\eta_{\text{p}}^{2}$ = 0.087], with low orthographic similarity items being responded to faster than high orthographic similarity items. There was no main effect of phonological similarity and no interaction (*p*'s > 0.1). In contrast, in the non-native accent, there was an interaction between phonology and orthography [*F*_1_(1,58) = 21.710, *p* < 0.001, $\eta_{\text{p}}^{2}$ = 0.272], such that, when orthography and phonology aligned (i.e., high similarity in both orthography and phonology or low similarity in both orthography and phonology), response times were reduced compared to the cases in which the two did not align (i.e., high phonological similarity but low orthographic similarity or vice-versa). There was also a marginal main effect of phonology [*F*_1_(1,58) = 3.352, *p* = 0.072, $\eta_{\text{p}}^{2}$ = 0.055], such that high phonological similarity items were responded to faster than low phonological similarity items. There was no main effect of orthographic similarity (*p* > 0.1). To summarize, in native speech, orthographic similarity led to slower processing of both high and low phonological similarity items. In non-native speech, the pattern was similar for low phonological similarity items—responded to slower in the case of high orthographic similarity, but the pattern was reversed for high phonological similarity—they were responded to faster in the case of higher orthographic similarity.

#### Phonological and Orthographic Similarity Effects on Signal Detection (A')

**Figure 3:**
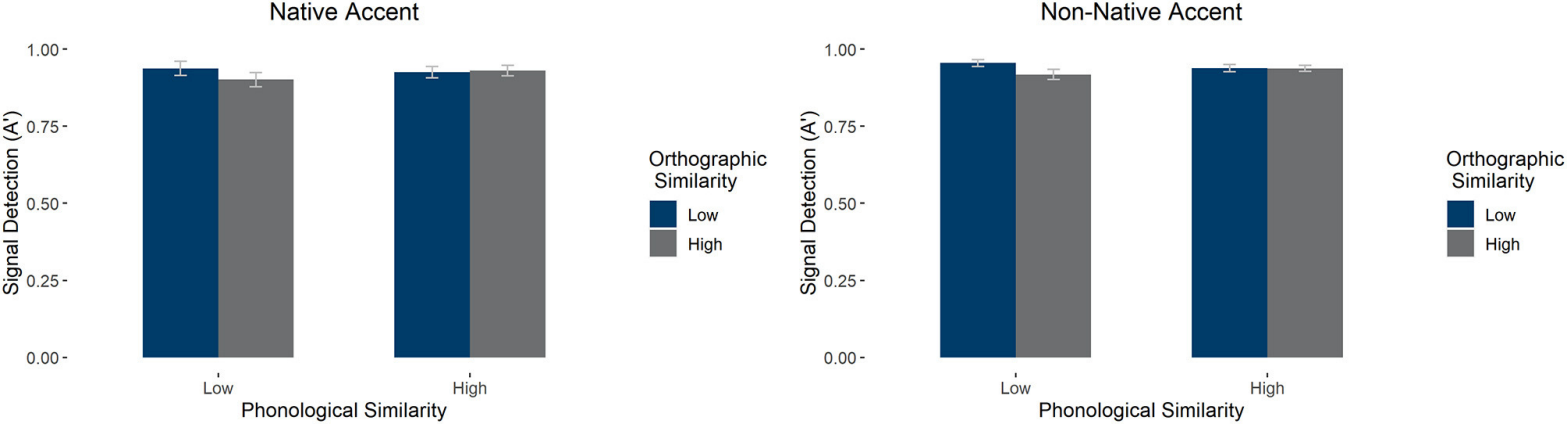
Average signal detection (as measured by A') in the LDT by accent, orthographic similarity, and phonological similarity condition. Error bars mark 95% confidence intervals.

We carried out an ANOVA on the effects of accent, phonological similarity, and orthographic similarity on signal detection. We found a main effect of orthography [*F*(1, 58) = 51.330, *p* < 0.001, $\eta_{\text{p}}^{2}$ = 0.469], qualified by an interaction between orthography and phonology [*F*(1, 58) = 55.249, *p* < 0.001, $\eta_{\text{p}}^{2}$ = 0.488]: Low phonological similarity items had higher signal detection when orthographic similarity was low, *F*(1, 58) = 87.100, *p* < 0.001, but this was not the case for high phonological similarity items, *F*(1, 58) =0.747, *p* = 0.391. See Figure 3 for average signal detection values (A'). There were no other main effects or interactions (*p*'s > 0.1).

#### Effects of Perfect Orthographic Cognates on Response Time

**Figure 4:**
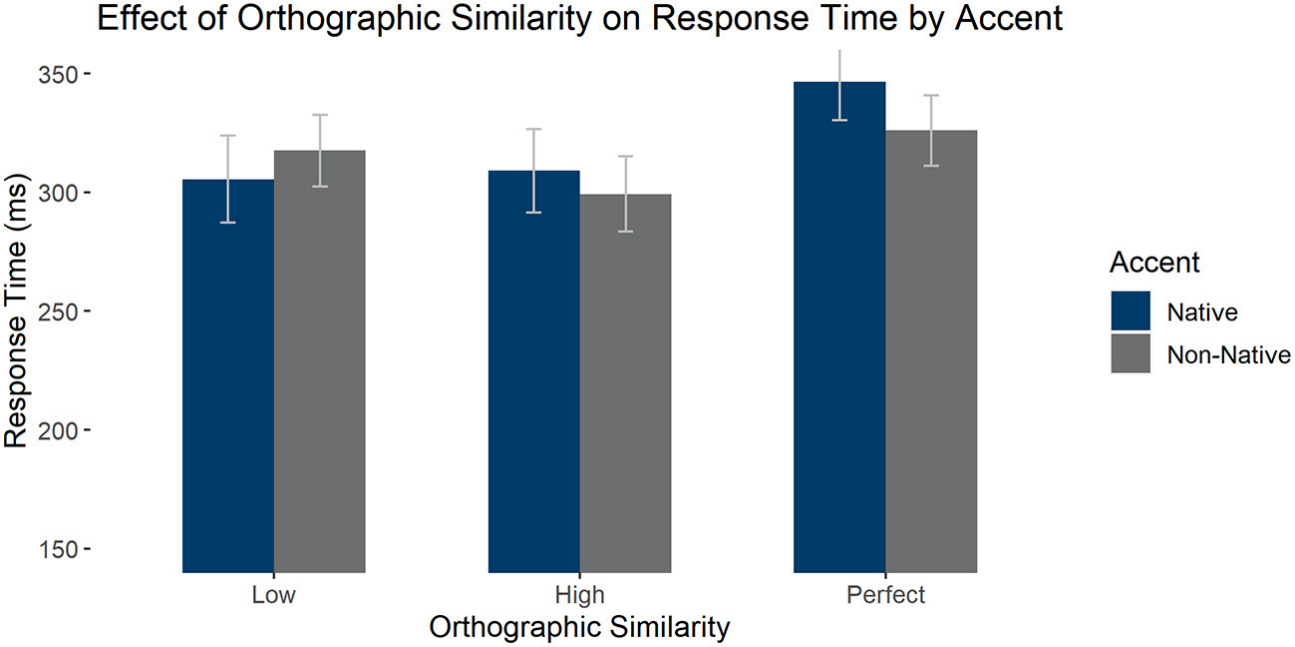
Average response times in the LDT to high phonological similarity words by accent and orthographic similarity condition. Error bars mark 95% confidence intervals.

We also analyzed the effect of perfect orthographic cognates and accent on response time, selecting only high phonological similarity items for the comparison: We compared high phonological similarity items that had low orthographic similarity, high orthographic similarity, or were perfect cognates. We found a main effect of orthographic similarity, *F*_1_(2, 116) = 36.774, *p* < 0.001, $\eta_{\text{p}}^{2}$ = 0.388 (absent by item *F*_2_(2, 147) = 1.858, *p* = 0.160, $\eta_{\text{p}}^{2}$ = 0.025), such that perfect cognates were responded to significantly slower than both high, *t*(58) = 8.169, *pholm* < 0.001, and low similarity items, *t*(58) = 6.346, *pholm* < 0.001, but the last two groups did not differ significantly, *t*(58) = 1.823, *pholm* = 0.071. There was an interaction between accent and orthography [*F*_1_(2, 116) = 14.818, *p* < 0.001, $\eta_{\text{p}}^{2}$ = 0.204; *F*_2_(1, 147) = 2.568, *p* = 0.080, $\eta_{\text{p}}^{2}$ = 0.034], such that there was an effect of accent only for the perfect cognates, *t*(58) = 2.954, *pholm* = 0.041, with participants responding slower in the native than the non-native accent. There was an effect of accent by item [*F*_1_(1, 58) = 0.886, *p* = 0.351, $\eta_{\text{p}}^{2}$ = 0.015; *F*_2_(1, 116) = 11.408, *p* < 0.001, $\eta_{\text{p}}^{2}$ = 0.072]. See Figure 4 for average response times by condition.

#### Effects of Perfect Orthographic Cognates on Signal Detection (A')

**Figure 5:**
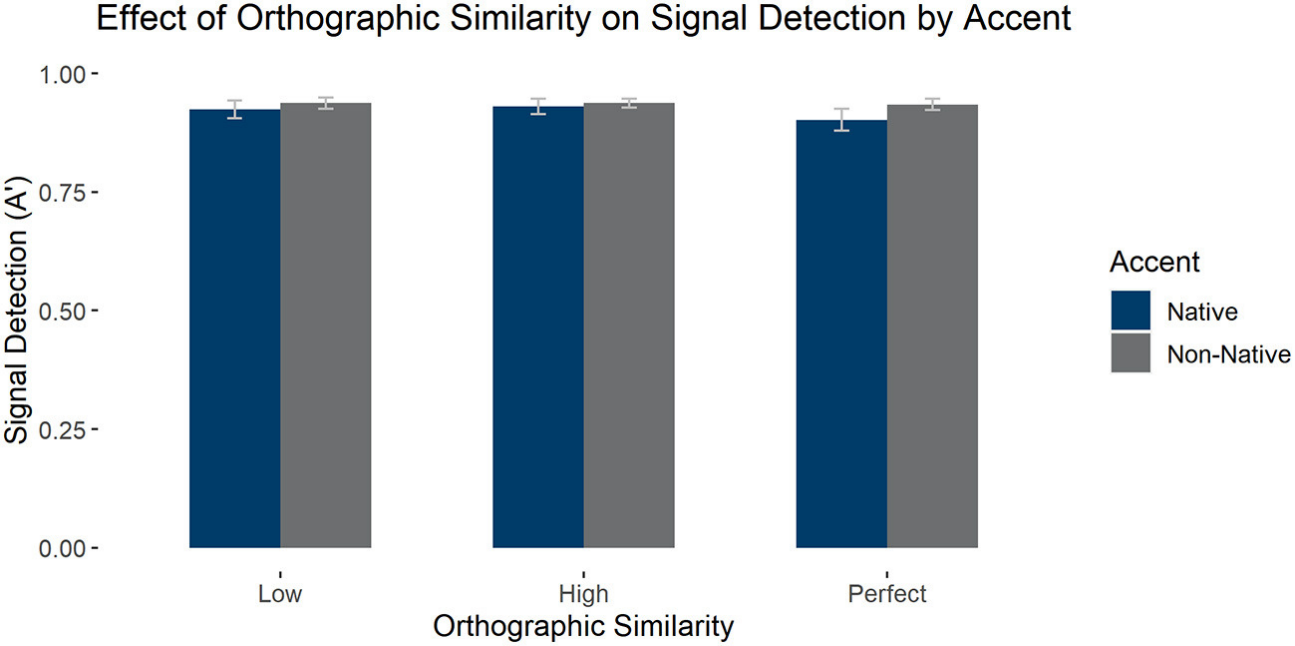
Average signal detection (as measured by A') in the LDT to high phonological similarity words by accent and orthographic similarity condition. Error bars mark 95% confidence intervals.

We also analyzed the effect of perfect orthographic cognates and accent on signal detection, selecting again only high phonological similarity items for the comparison. We found a marginal main effect of accent [*F*(1, 58) = 3.960, *p* = 0.051, $\eta_{\text{p}}^{2}$ = 0.064], such that there was higher signal detection with the non-native accent, as well as a main effect of orthographic similarity [*F*(1, 58) = 8.738, *p* < 0.001, $\eta_{\text{p}}^{2}$ = 0.131], such that signal detection was significantly worse for perfect cognates than both high [*t*(58) = 3.516, *pholm* = 0.003] and low similarity items [*t*(58) = 3.024, *pholm* = 0.007], but high and low similarity items did not differ, *p* > 0.1. This effect was qualified by an interaction between accent and orthographic similarity, *F*(1, 58) = 6.855, *p* = 0.002, $\eta_{\text{p}}^{2}$ = 0.106, such that the effect of orthographic similarity was only present in the native accent [*F*(1, 58) = 11.442, *p* < 0.001, $\eta_{\text{p}}^{2}$ = 0.165] but not in the non-native accent, *p* > 0.1. See Figure 5 for average signal detection scores by group.

To summarize, considering response times and signal detection together, there was a strong disadvantage in processing of high phonological similarity items when orthographic similarity was high (slower and less accurate). Importantly, this was predominantly present in the native accent.

### Typing Task

#### Phonological and Orthographic Similarity Effects on Accuracy

**Figure 6:**
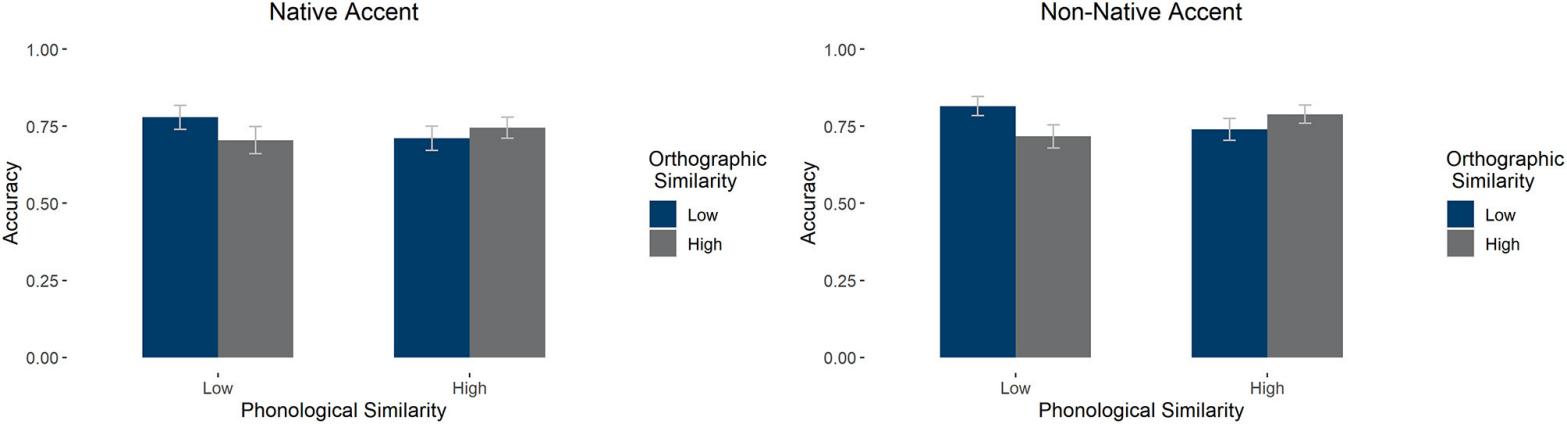
Average accuracy in the typing task by accent, phonological similarity, and orthographic similarity condition. Error bars mark 95% confidence intervals.

There was a main effect of accent—with participants showing higher accuracy in the non-native condition [*F*_1_(1,57) = 15.946, *p* < 0.001, $\eta_{\text{p}}^{2}$ = 0.219; *F*_2_(1, 196) = 9.952, *p* = 0.002, $\eta_{\text{p}}^{2}$ = 0.048] and orthographic similarity—with low similarity items leading to higher accuracy [*F*_1_(1,57) = 23.845, *p* < 0.001, $\eta_{\text{p}}^{2}$ = 0.295, absent by item *F*_2_(1, 196) = 0.639, *p* =0.425, $\eta_{\text{p}}^{2}$ = 0.003]. There was a significant interaction between phonological and orthographic similarity [*F*_1_(1,57) = 122.28, *p* < 0.001, $\eta_{\text{p}}^{2}$ = 0.682; *F*_2_(1, 196) = 5.202, *p* = 0.024, $\eta_{\text{p}}^{2}$ = 0.026]. This interaction showed that, in both accents, accuracy was greater when orthographic and phonological similarity aligned: Participants were better at typing (i.e., identifying the correct word) when the words were high phonological and orthographic similarity items or low phonological and orthographic similarity items as compared to high phonological and low orthographic similarity items or low phonological and high orthographic similarity items. There was also a marginal interaction between accent and phonological similarity [*F*_1_(1,57) = 3.283, *p* = 0.075, $\eta_{\text{p}}^{2}$ = 0.055, absent by item *F*_2_(1, 196) = 0.408, *p* = 0.524, $\eta_{\text{p}}^{2}$ = 0.002] and a three-way interaction [*F*_1_(1,57) = 7.303, *p* = 0.009, $\eta_{\text{p}}^{2}$ = 0.114, absent by item *F*_2_(1, 196) = 1.024, *p* = 0.313, $\eta_{\text{p}}^{2}$ = 0.005]. Follow-up simple comparisons on the three-way interaction showed that there was a significant positive effect of non-native accent in all cases [high orthographic and phonological similarity: *t*(57) = 4.89, *pholm* < 0.001; low orthographic and high phonological similarity: *t*(57) = 3.13, *pholm* = 0.025, and low orthographic and phonological similarity: *t*(57) = 3.40, *pholm* = 0.014] except for low phonological and high orthographic similarity items, where there was no effect [*t*(57) = 1.22, *pholm* = 1]. There was no main effect of phonological similarity, nor an interaction between accent and orthographic similarity, *p*'s > 0.1. See Figure 6 for average accuracy by group.

#### The Effects of Perfect Orthographic Cognates on Accuracy

**Figure 7:**
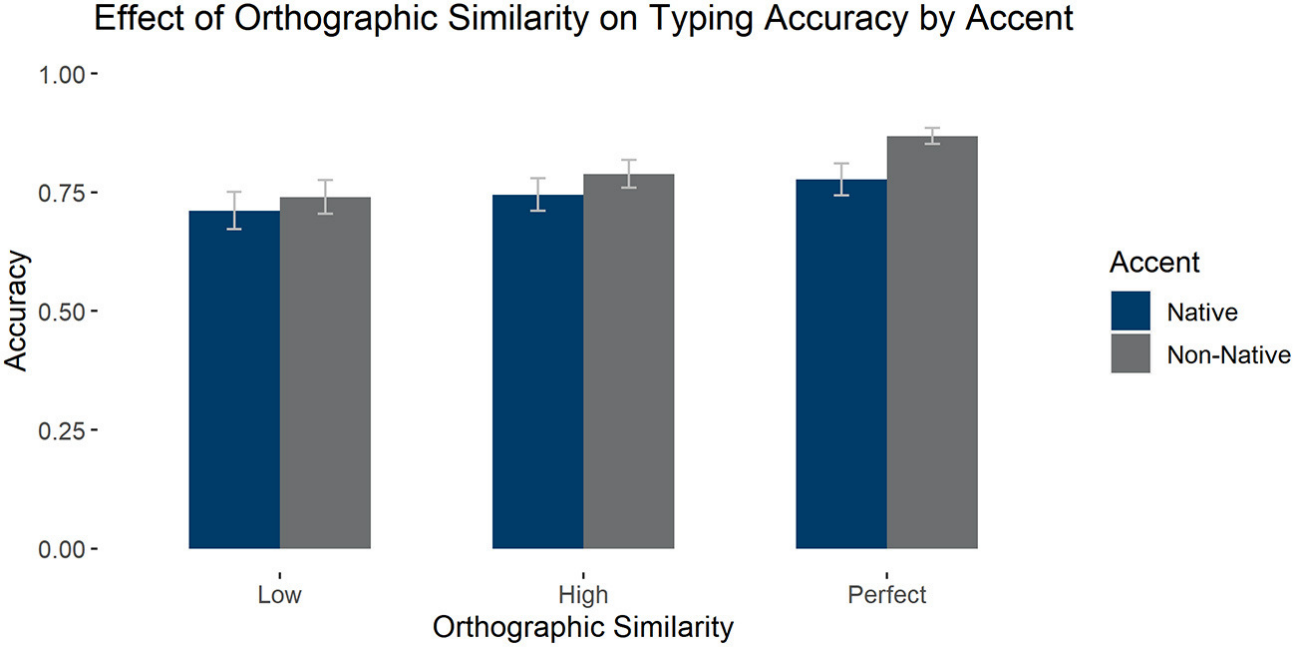
Average accuracy in the typing task for high phonological similarity words by accent and orthographic similarity condition. Error bars mark 95% confidence intervals.

We also analyzed the effect of perfect orthographic cognates and accent on accuracy, selecting only high phonological similarity items for the comparison. We found a main effect of accent—such that the non-native accent led to higher accuracy [*F*_1_(1,57) = 48.673, *p* < 0.001, $\eta_{\text{p}}^{2}$ = 0.461; *F*_2_(1, 147) = 18.151, *p* < 0.001, $\eta_{\text{p}}^{2}$ = 0.110] and of orthographic similarity [*F*_1_(2,114) = 58.019, *p* < 0.001, $\eta_{\text{p}}^{2}$ = 0.504; *F*_2_(2, 147) = 3.071, *p* = 0.049, $\eta_{\text{p}}^{2}$ = 0.040], such that perfect cognates were identified more accurately than high [*t*(57) = 6.530, *pholm* < 0.001] and low similarity items [*t*(57) = 8.809, *pholm* < 0.001], while high similarity items were identified better than low similarity items [*t*(57) = 5.779, *pholm* < 0.001]. There was an interaction between accent and orthography [*F*_1_(2,114) = 19.613, *p* < 0.001, $\eta_{\text{p}}^{2}$ = 0.256, absent by item *F*_2_(2, 147) = 2.144, *p* = 0.121, $\eta_{\text{p}}^{2}$ = 0.028] such that the effects of accent were greater in the perfect condition than in the other two. See Figure 7 for average accuracy by group.

Overall for the typing task looking at orthographic and phonological similarity categorically, we find that the non-native accent aided performance, particularly in the case of perfect cognates. Furthermore, orthographic and phonological similarity interact such that words for which both types of similarity are aligned are typed more accurately than those for which the two are crossed.

## Discussion

The current study set out to explain the inhibitory effect of orthographic similarity on auditory word recognition in bilinguals. In a previous study, we showed that orthographic similarity had an inhibitory effect in the auditory modality (Frances et al., 2021). This is in contrast to the facilitatory cognate effect that has been found in the visual modality (Caramazza and Brones, 1979; Cristoffanini et al., 1986; de Groot and Nas, 1991; Sanchez-Casas et al., 1992; Dijkstra et al., 1998, 1999; Schwartz et al., 2007; Voga and Grainger, 2007; but see Schwartz et al., 2007). It should be noted that even though the definition of cognate refers to similarity in form (which should also include the phonological form), they are generally defined orthographically—meaning by similarity in spelling—and studied in the visual modality (Caramazza and Brones, 1979; Sanchez-Casas et al., 1992; Dijkstra et al., 1998; Colom, 2001; Zhang and Mueller, 2005; but see Dijkstra et al., 1999; Schwartz et al., 2007). Our current study finds inhibitory effects of orthographic similarity in the auditory modality and aligns with the results of Frances et al. (2021). Importantly, in our case, we took a step further and tried to understand the origin of this effect—namely, inhibition in processing of orthographically similar words in the auditory modality.

This study explored two alternative hypotheses to explain this effect. One possibility was that the listener's phonological representation of an item in the FL differed from the native production *more* in cases of higher orthographic similarity. Another possibility we explored was that listeners were incorrectly “transcribing” the FL items they heard using their NL orthographic rules (as opposed to their FL rules). Both cases would lead to slower response times and worse identification of items with higher orthographic similarity to their NL when participants were exposed to native accented speech (Frances et al., 2021). Therefore, to test and disambiguate these two possibilities, we had participants carry out both an LDT and a typing task presenting the stimuli in the auditory modality. The items we presented were produced with either a native or a non-native accent (similar to the participants'). If the first hypothesis were true, we would expect that the non-native accent would reduce the discrepancy between the internal representation and the exemplar heard, thus reducing the inhibitory effect of orthographic similarity in the LDT. Furthermore, the orthographic similarity effect should revert and be facilitatory in the typing task. If the second hypothesis were true, we would expect no difference between accents in the LDT, and we would see a detrimental effect of orthographic similarity in the typing task as well as the LDT.

As expected and in replication to Frances et al. (2021), orthographic similarity had a negative effect on signal detection in the auditory LDT, which extended to accuracy in the typing task. For the LDT, we found that using a non-native accent reduced response times and that orthographic similarity was particularly detrimental in cases of high phonological similarity—both in response times and accuracy—specifically in the native accent. We also found that the effect of accent was disproportionately larger for perfect cognates than for high orthographic similarity items. When considering similarity linearly, we found orthographic (inhibitory) and phonological (facilitatory) effects on accuracy. We also found that phonological similarity had the largest effects for words that were more orthographically similar and presented in the non-native accent—matching that of the listener. In the typing task, we also found higher accuracy in the non-native accent condition, particularly in the case of perfect cognates. Furthermore, when similarity was high or low for both orthography and phonology, accuracy was higher than when they were crossed.

The disproportionate effects we found of perfect cognates on both response time (LDT) and word recognition (accuracy in the typing task) align with prior studies that suggest that perfect cognates (or identical cognates) have a “special status” for bilinguals (Dijkstra et al., 1999; Lemhöfer and Dijkstra, 2004). This suggests that sharing the same orthographic item between two languages creates confusion and difficulties in the auditory modality. It is possible that this effect extends to lexical representation in general (i.e., not just orthographic, but also phonological representations), but to establish the extent of this effect, we would need to test perfect phonological cognates. Unfortunately, this was not possible within our study due to the language combination we addressed, which favored a relative dissociation between phonological and orthographic similarity. Therefore, this specific group of words—namely, perfect phonological cognates—should also be tested, as well as languages that share more of their phonemes and phoneme to grapheme correspondences. Finally, we found that accent facilitated word recognition and detection, as has been suggested in other studies (Bent and Bradlow, 2003; Xie and Fowler, 2013; Wang and van Heuven, 2015). This has been referred to as the inter language speech intelligibility benefit (Bent and Bradlow, 2003; Xie and Fowler, 2013; Wang and van Heuven, 2015). Studies so far have mostly focused on overall intelligibility and sentence comprehension, but we were able to extend these results to the word level and show that this effect interacts with other variables, such as orthography.

With respect to our original hypotheses, we found a reduction of the inhibitory orthographic effect in response time with the non-native accent and increased accuracy with the non-native accent, with both results supporting our first hypothesis—namely, a discrepancy in the FL phonological representation. In support of our second hypothesis, accuracy in both the LDT and the typing task showed the same inhibitory effects of orthography for both accent conditions. Overall, we can say our second hypothesis was more strongly supported, but there is evidence that the alignment between the phonological representation of the FL item and the specific auditory stimulus does play a role in auditory processing. In practical terms, hearing words in a non-native accent matching that of the listener seems to help both identify and spell the word more accurately, but the differences in phoneme to grapheme correspondences between the NL and FL make word identification and spelling more difficult. This would also mean that, when hearing words in an FL, participants attempt to “transcribe” these items and orthographic similarity between one's NL and FL becomes confusing.

One important limitation of our study is that we cannot speak to the possible effects of stronger or weaker accents or different regional accents, since we compared only two specific voices. Nevertheless, to our knowledge, there are no prior studies looking at the effects of accent in cognate processing—particularly perception—and the only prior study looking at the auditory effects of cognates is Frances et al. (2021). Furthermore, we were able to observe orthographic and phonological similarity effects separately—which also had not been done before—and tease apart the effects of each. Even though we focused on one pair of languages—namely, English and Spanish—our study highlights that orthographic and phonological similarity do not necessarily have the same effects. Indirectly, this points to the importance of the relationship between the languages of a bilingual when studying the interaction of visual and auditory representations—i.e., orthography and phonology—in processing. In other words, in languages with contradicting phoneme to grapheme conversion rules these factors are likely to have different effects on processing than in language combinations that have different writing systems (e.g., Greek and English or, even more so, Mandarin and English) or very similar phoneme to grapheme conversion rules (e.g., Spanish and Basque or Spanish and Italian). This, as well as the distinction between orthographic and phonological effects, is not contemplated so far in bilingual language processing models (Dijkstra and van Heuven, 2002; Brysbaert and Duyck, 2010). Our results suggest that it is necessary to integrate orthography and phonology as well as the relationship between languages (i.e., similarities and differences between them at various levels) into our current models of bilingual language processing. In the case of the auditory modality, it is also important to integrate “external” or “environmental” factors, so to speak, such as accent (as shown in the present study) or noise (see (Guediche et al., 2021; Navarra-Barindelli et al., 2021) showing a reduction of the cognate effect in noise) that are unique to each specific instance of the auditory input. More specifically in reference to our work, it is important to not only think of the phonology or phonological representations but also of the particular phonetics of the auditory stimulus.

Although this does not diminish the relevance of the effects we found, it is important to test other language combinations in order to fully understand the interactions between the languages of a bilingual. As a whole, our results suggest that the inhibitory orthographic similarity effect in auditory word perception in bilinguals is at least partially due to the relationship between the languages—their orthographies and opacity or transparency—as well as to whether the item is produced more differently or more similarly to the listener's own accent. Our results also call for more complex models of language processing that take into account different modalities and the relationship between the languages of a bilingual. In other words, we cannot expect the same effects when reading or listening and we cannot expect the same effects in Mandarin/English bilinguals as in Spanish/English or Spanish/Basque bilinguals. Future studies should focus on expanding these results to other sets of languages in order to assess the role of the relationship between languages in the effects of orthographic and phonological similarity.

## Conclusions

In the current study, we found that both the accent in which an item is produced and the phoneme to grapheme conversion rules of the FL modulate the effect of orthographic similarity on auditory word processing. In general, detecting whether a phonological string is a word (LDT) was not affected by accent, but spelling out the correct word (typing task) was. Orthographic similarity had a negative effect in both cases and phonological similarity improved accuracy in the typing task, but not the LDT. Although further studies are needed in order to fully elucidate the origin of the inhibitory effect of orthographic similarity in the auditory modality, our results have both theoretical—pointing toward the need to take different modalities and language combinations into account for bilingual language processing models—as well as practical implications—for example, for foreign language learning.

## Data Availability Statement

The datasets presented in this study can be found in online repositories. The names of the repository/repositories and accession number(s) can be found at: https://osf.io/pr3es/?view_only=affcb60dc99b48868df05bcf68f2e671.

## Ethics Statement

The studies involving human participants were reviewed and approved by Basque Center on Cognition, Brain and Language Ethics Committee. The patients/participants provided their written informed consent to participate in this study.

## Author Contributions

CF, EN-B, and CM all conceived of the idea. CF and EN-B designed the experiment. EN-B carried out the experiment. CF programmed the experiment, wrote the manuscript, and did the analyses. CM supervised the project. The final manuscript was reviewed and commented by all authors.

## Funding

This research was supported by the Basque Government through the BERC 2022-2025 program and by the Spanish State Research Agency through BCBL Severo Ochoa excellence accreditation CEX2020-001010-S. CF and EN-B are supported by MINECO predoctoral grants from the Spanish government (BES-2016-077169) and (BES-2016-078896) respectively. CM was further supported by the Spanish Ministry of Economy and Competitiveness [PID2020-113926GB-I00, PSI2017-82941-P, and RED2018-102615-T] and the Basque Government [PIBA18-29] and funding from the European Research Council (ERC) under the European Union's Horizon 2020 research and innovation programme (Grant Agreement No:819093 to CM).

## Conflict of Interest

The authors declare that the research was conducted in the absence of any commercial or financial relationships that could be construed as a potential conflict of interest.

## Publisher's Note

All claims expressed in this article are solely those of the authors and do not necessarily represent those of their affiliated organizations, or those of the publisher, the editors and the reviewers. Any product that may be evaluated in this article, or claim that may be made by its manufacturer, is not guaranteed or endorsed by the publisher.
