# Supplementary material for: Speaker Accent Modulates the Effects of Orthographic and Phonological Similarity on Auditory Processing by Learners of English
Source: Front Psychol. 2022 May 19;13:892822. doi: 10.3389/fpsyg.2022.892822 (PMC9161262; doi:10.3389/fpsyg.2022.892822)
Supplement: Supplementary file 1 [file Table_1.DOCX]

**Supplementary Materials**

1. **Analysis**
   1. **Lexical decision task**

Although the binary measures of orthographic and phonological similarity provide a good, orthogonal measure of these two types of similarity, in order to properly match the conditions, the range of similarity values had to be restricted. In addition, because these two measures—orthographic and phonological similarity—are strongly correlated, the mismatch groups (i.e., high orthographic similarity but low phonological similarity and low orthographic similarity but high phonological similarity groups) might be composed of items that are somehow unique. The mixed models were included as an extension in order to compensate for these possible pitfalls and as a way to assess the effects of phonological and orthographic similarity in a linear way using the full spectrum of values. The first model had the logarithmic transformation of response time as the dependent variable and the second had accuracy (correct/incorrect) as the dependent variable. The fixed effects in both cases were accent (native or non-native), phonological similarity (as defined by ALINE distance), and orthographic similarity (as defined by ALINE distance) as well as their interactions. In addition, we included the random intercepts and slopes for accent by subject and item. This model converged and the results were interpretable. Given the special status of perfect cognates, they were excluded from these analyses.

ALINE distances were calculated using the alineR package for R [47]. ANOVAs were run using JASP [48] and mixed models were run using the lme4 [49] and lmerTest [50] packages in R [51,52].

- 1. **Typing task**

For this task, we carried out the same analysis as with the LDT: a mixed effect model with accuracy as the dependent variable. The model had accuracy (1 if a correct or 0 if an incorrect word produced) as the dependent variable and accent (native or non-native), phonological similarity (as defined by ALINE distance), and orthographic similarity (as defined by ALINE distance) as well as their interactions as fixed effects. In addition, we included the random intercepts and slopes for accent by subject and item. This model converged and the results were interpretable. Given the special status of perfect cognates, they were excluded from this analysis. The analysis was also run as linear models using ALINE distance [47] instead of the binary accuracy. The pattern and significant effects and interaction were strictly identical.

1. **Results**
   1. **Lexical Decision Task**
      1. **The effects of phonological and orthographic similarity as continuous variables on response time**

In the model, there was a significant main effect of accent [*β* = .041, *SE* = .011, *t*(187.9) = 3.728, *p* < .001] with participants responding slower to the non-native than the native accent (*Note:* given that the speech rates were not matched between speakers, between-speaker comparisons are not reliable). There was no main effect of phonological similarity [*β* = -.117, *SE* = .080, *t*(246.3) = -1.470, *p* = .143] or orthographic similarity [*β* = .050, *SE* = .069, *t*(246.2) = .714, *p* = .476] and no interactions between phonological similarity and accent [*β* = -.021, *SE* = .059, *t*(251) = -.352, *p* = .725], orthographic similarity and accent [*β* = -.011, *SE* = .051, *t*(250.7) = -.214, *p* = .831], or phonological and orthographic similarity [*β* = .101, *SE* = .206, *t*(245.9) = .492, *p* = .623]. There was also no three-way interaction [*β* = -.249, *SE* = .152, *t*(249) = -1.637, *p* = .103]. See Figure S.1 for regression lines of response times by group.


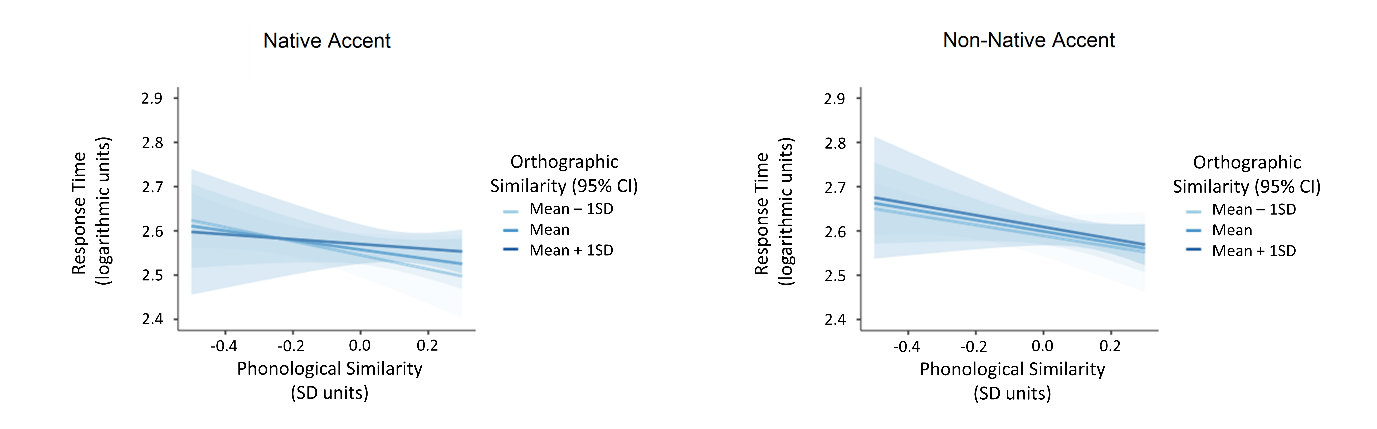


Figure S.1: Regression lines for response times in the LDT by the continuous measures of phonological and orthographic similarity by accent.

- - 1. **The effects of phonological and orthographic similarity as continuous variables on accuracy**

In the model, there was a significant main effect of phonological similarity—with higher phonological similarity leading to higher accuracy [*β* = 2.163, *SE* = .899, *z* = 2.406, *p* = .016], as well as orthographic similarity—with higher orthographic similarity leading to lower accuracy [*β* = -1.724, *SE* = .785, *z* = -2.195, *p* = .028], and a marginal three-way interaction—such that the effect of phonological similarity was greater for high orthographic similarity items in the non-native condition [*β* = 3.606, *SE* = 2.143, *z* = 1.683, *p* = .092]. There was no main effect of accent [*β* = .024, *SE* = .159, *z* = .150, *p* = .881] and no interactions between phonological similarity and accent [*β* = .801, *SE* = .837, *z* = .957, *p* = .339], orthographic similarity and accent [*β* = -.959, *SE* = .730, *z* = -1.314, *p* = .189], or phonological and orthographic similarity [*β* = -.715, *SE* = 2.251, *z* = -.318, *p* = .751]. See Figure S.2 for regression curves of accuracy by group.

In sum, considering orthographic similarity and phonological similarity as linear variables, we found that there are orthographic (inhibitory) and phonological (facilitatory) similarity effects on accuracy, which interact such that the largest effects of phonological similarity occur for high orthographic similarity items in the non-native condition. In other words, when the stimuli are presented with a non-native accent matching that of the listener, phonological similarity is particularly helpful in the case of high orthographic similarity.


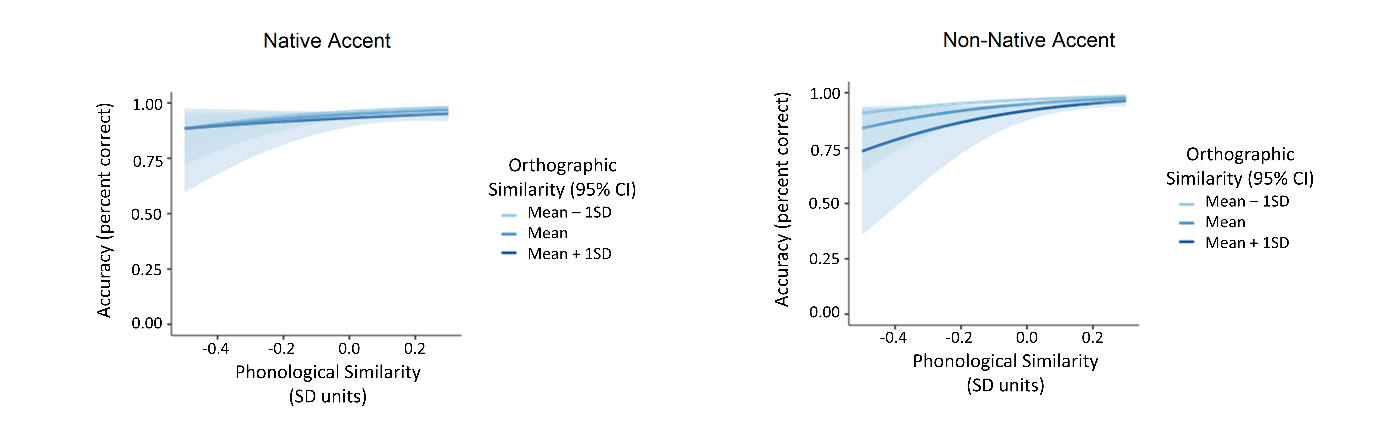


Figure S.2: Regression curves for accuracy in the LDT by the continuous measures of phonological and orthographic similarity by accent.

- 1. **Typing Task**
     1. **The effects of phonological and orthographic similarity as continuous variables on accuracy**

In the model, there was a significant main effect of phonological similarity—with higher phonological similarity leading to higher accuracy [*β* = 2.169, *SE* = 1.059, *z* = 2.047, *p* = .041], as well as orthographic similarity—with higher orthographic similarity leading to lower accuracy [*β* = -1.859, *SE* = .927, *z* = -2.006, *p* = .045], and a three-way interaction—such that the effect of phonological similarity was greater for high orthographic similarity items in the non-native condition [*β* = 3.915, *SE* = 1.818, *z* = 2.154, *p* = .031]. There was no main effect of accent [*β* = .182, *SE* = .114, *z* = 1.601, *p* = .109] and no interactions between phonological similarity and accent [*β* = .319, *SE* = .718, *z* = .445, *p* = .656], orthographic similarity and accent [*β* = -.193, *SE* = .627, *z* = -.308, *p* = .758], or phonological and orthographic similarity [*β* = 1.698, *SE* = 2.667, *z* = .637, *p* = .524]. See Figure S.3 for regression lines of accuracy by group.

Considering orthographic and phonological similarity as linear variables, we again see that for the non-native accent, phonological similarity is particularly helpful in the case of high orthographic similarity.


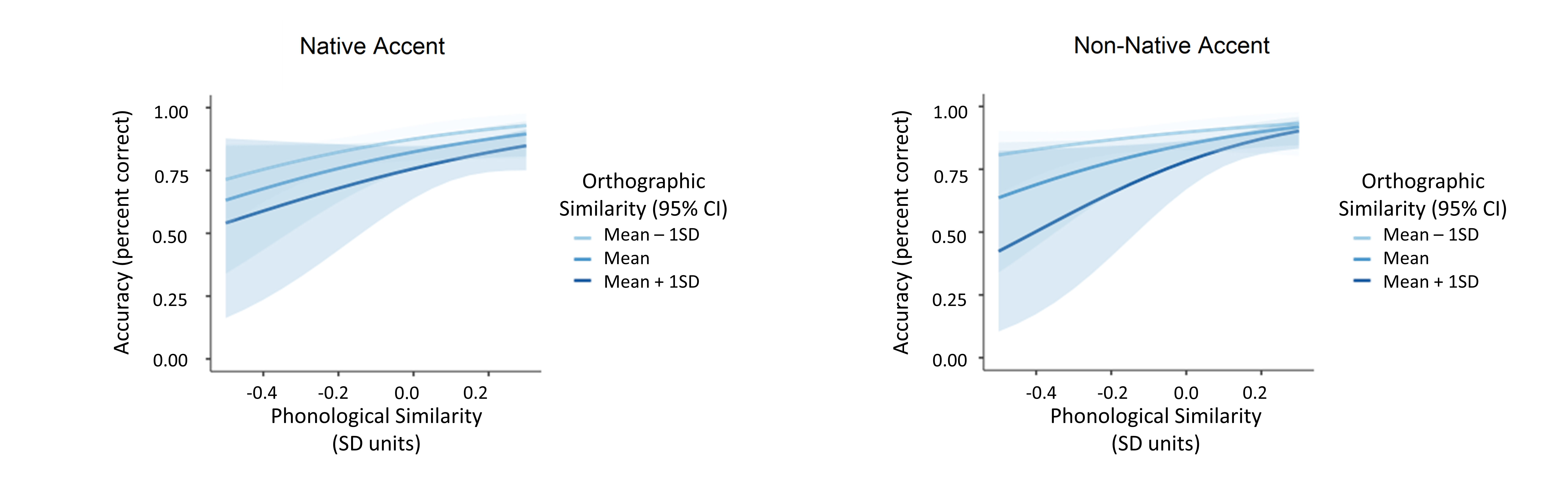


Figure S.3: Regression curves for average accuracy in the typing task by the continuous measures of phonological and orthographic similarity by accent.
